# Predictive modelling for late rectal and urinary toxicities after prostate radiotherapy using planned and delivered dose

**DOI:** 10.3389/fonc.2022.1084311

**Published:** 2022-12-16

**Authors:** Ashley Li Kuan Ong, Kellie Knight, Vanessa Panettieri, Mathew Dimmock, Jeffrey Kit Loong Tuan, Hong Qi Tan, Caroline Wright

**Affiliations:** ^1^ Division of Radiation Oncology, National Cancer Centre, Singapore, Singapore; ^2^ Medical Imaging and Radiation Sciences, Monash University, Clayton, VIC, Australia; ^3^ Alfred Health Radiation Oncology, Alfred Hospital, Melbourne, VIC, Australia; ^4^ School of Allied Health Professions, Keele University, Staffordshire, United Kingdom

**Keywords:** normal tissue complication probability (NTCP), Lyman-Kutcher-Burman (LKB) model, accumulated dose, high-risk prostate cancer, image-guided radiotherapy (IGRT), late toxicity complications

## Abstract

**Background and purpose:**

Normal tissue complication probability (NTCP) parameters derived from traditional 3D plans may not be ideal in defining toxicity outcomes for modern radiotherapy techniques. This study aimed to derive parameters of the Lyman-Kutcher-Burman (LKB) NTCP model using prospectively scored clinical data for late gastrointestinal (GI) and genitourinary (GU) toxicities for high-risk prostate cancer patients treated using volumetric-modulated-arc-therapy (VMAT). Dose-volume-histograms (DVH) extracted from planned (D_P_) and accumulated dose (D_A_) were used.

**Material and methods:**

D_P_ and D_A_ obtained from the DVH of 150 prostate cancer patients with pelvic-lymph-nodes irradiation treated using VMAT were used to generate LKB-NTCP parameters using maximum likelihood estimations. Defined GI and GU toxicities were recorded up to 3-years post RT follow-up. Model performance was measured using Hosmer-Lemeshow goodness of fit test and the mean area under the receiver operating characteristics curve (AUC). Bootstrapping method was used for internal validation.

**Results:**

For mild-severe (Grade ≥1) GI toxicity, the model generated similar parameters based on D_A_ and D_P_ DVH data (D_A_-D_50_:71.6 Gy vs D_P_-D_50_:73.4; D_A_-m:0.17 vs D_P_-m:0.19 and D_A/P_-n 0.04). The 95% CI for D_A_-D_50_ was narrower and achieved an AUC of >0.6. For moderate-severe (Grade ≥2) GI toxicity, D_A_-D_50_ parameter was higher and had a narrower 95% CI (D_A_-D_50_:77.9 Gy, 95% CI:76.4-79.6 Gy vs D_P_-D_50_:74.6, 95% CI:69.1-85.4 Gy) with good model performance (AUC>0.7). For Grade ≥1 late GU toxicity, D_50_ and n parameters for D_A_ and D_P_ were similar (D_A_-D_50_: 58.8 Gy vs D_P_-D_50_: 59.5 Gy; D_A_-n: 0.21 vs D_P_-n: 0.19) with a low AUC of<0.6. For Grade ≥2 late GU toxicity, similar NTCP parameters were attained from D_A_ and D_P_ DVH data (D_A_-D_50_:81.7 Gy vs D_P_-D_50_:81.9 Gy; D_A_-n:0.12 vs D_P_-n:0.14) with an acceptable AUCs of >0.6.

**Conclusions:**

The achieved NTCP parameters using modern RT techniques and accounting for organ motion differs from QUANTEC reported parameters. D_A_-D_50_ of 77.9 Gy for GI and D_A_/D_P_-D_50_ of 81.7-81.9 Gy for GU demonstrated good predictability in determining the risk of Grade ≥2 toxicities especially for GI derived D_50_ and are recommended to incorporate as part of the DV planning constraints to guide dose escalation strategies while minimising the risk of toxicity.

## Introduction

External beam radiotherapy (RT) plays an important role in the clinical management of patients with locally advanced high-risk prostate cancer (HR-PCa) (1). Improvements in biochemical disease-free survival and overall survival in patients with HR-PCa have been reported to correlate with higher radiation doses delivered in a hypofractionated manner (2). The low alpha-beta ratio (α/β= 1.5 -3 Gy) of the prostate cancer cells, similar to that of the late responding normal tissues can be attributed to this unique phenomenon (3, 4). However, the prescription of a higher radiation dose is often associated with an increased risk of radiation-induced toxicity (5, 6). To better estimate the impact of prescribing a higher RT dose on the toxicity risk, the use of biological predictive models will have a higher relevance as compared to the conventional method of using physical dose-volume (DV) values (7). Biological models can account for different treatment fractionations, overall treatment duration and tumour sensitivity (8).

To date, most of the DV values and radiobiological parameters are based on QUANTEC (quantitative analysis of normal tissue effects in the clinic) recommendations (9, 10). Dose-response parameters derived from QUANTEC have been obtained from plans generated using three-dimensional conformal radiotherapy techniques (11). The use of modern radiotherapy (RT) planning techniques (e.g. volumetric modulated arc therapy (VMAT) and intensity-modulated RT (IMRT) results in significant variations in dose distributions to the targets and proximal organs at risk (OARs) (12). The high dose conformality to the target achieved using VMAT/IMRT and a corresponding reduction in dose to the OARs changes the toxicity profiles for this group of patients (13, 14). As such, the incorporation of the most widely used dose-based Lyman-Kutcher-Burman (LKB) model (based on modern planning techniques) has an advantage over the use of standard DV metrics; this advantage manifests as the entire dose range are considered in predicting potential toxicity (15). Furthermore, by incorporating the variations in dose distribution due to interfractional organ motion, the parameters derived from LKB-NTCP modelling typically have a higher predictive power in estimating the potential clinically acceptable GI and GU complication rates while performing dose escalation strategies (16).

However, there is a paucity of published recommendations on LKB-NTCP parameters attained from the use of modern techniques such as VMAT/IMRT in predicting late GI and GU toxicities in HR-PCa (17). Additionally, it has been reported that it is technically challenging and resource-intensive to devise dose accumulation workflows and streamline this into existing work processes in busy RT clinics (18). In this study, we hypothesise that LKB-NTCP parameters generated from dose-volume-histograms (DVHs) from accumulated-dose (D_A_) data are superior in predicting late GI and GU toxicities as compared to those derived from planned-dose (D_P_) data.

This study aimed to estimate the NTCP parameters using an LKB model based on prospectively recorded toxicity data from patients treated with VMAT to predict late GI and GU toxicities at three years post-RT follow-up. Model performances of the derived parameters generated using either D_P_ or D_A_ to predict the defined toxicity were evaluated.

## Material and methods

### Patients and treatment

This study recruited 150 patients with localized HR-PCa who were treated with RT at the National Cancer Centre Singapore from 2016 to 2020. The median follow-up-time (FUT) for the entire cohort was 57 months, ranging from 31.8 to 77.0 months. Approval was obtained for this retrospective study from the centralized institutional review board (CIRB ref: 2019/2018). All patients were treated using the VMAT technique with a 10 MV photon energy. The RT dose prescription comprised of 46-54 Gy (2 Gy per fraction) to the prostate and pelvic lymph nodes for phase-1 followed by a sequential coned down phase-2 treatment dose of 24-28 Gy delivered to a volume that encompassed the prostate and proximal 1 cm of the seminal vesicles.

### Clinical endpoints

For each patient, late GI and GU toxicities were recorded at post-RT FUT of three months, and then every six months up to five years, and then yearly after that. In this study, late toxicity was defined as the maximum score recorded at the post-RT FUT of three months. Additionally, a post RT FUT cut-off at three years was applied for this analysis.

### Dosimetric data

Two types of dosimetric data from each patient were analysed: D_P_ and D_A_. For a given patient, the D_P_ value obtained from the DVH of the patient’s RT plan whereas the D_A_ value was extracted from the DVH generated from the previously developed dose accumulation workflow (19, 20). D_A_ accounted for the patient’s inter-fractional organ variations based on the daily acquired cone-beam computed tomography images.

### NTCP modelling

An LKB-NTCP model was employed in this study for the fitting of biological parameters based on the defined clinical toxicity data. The DV metrics were extracted from the D_P_ and D_A_ data respectively, where the details on the generation of D_A_ values have been previously described (21).

The LKB-NTCP model with a generalized equivalent uniform dose (gEUD) formulation was utilized and calculated as (22).


(1) $$NTCP_{LKB}=\frac{1}{\sqrt{2\pi }}\int_{-\infty }^{t}\exp\left(-\frac{x^{2}}{2}\right)dx$$

where,


(2) $$t=\frac{\text{gEUD}-D_{50}}{mD_{50}}$$

and,


(3) gEUD = (∑i=1Ividi1n)n

In Equations 1-3, gEUD is the sum over *I* bins in the DVH histogram, *D_50_* is the value of the uniform dose delivered to the entire organ that relates to a 50% complication probability, *m* is a derived quantity that is inversely proportional to the slope of the dose-response curve, and *n* parameterises the volume effect of the organ. Small values of *n* indicate sensitivity to high dose regions whereas values closer to 1 indicate that the response is due to average effect across the organ (17).

### Statistical analysis

The maximum likelihood estimation was used to find the best-fit parameters for LKB-NTCP. For a datapoint *k* of a given triplet parameters (D_50_, m, n), the logarithm of the likelihood (LLH) of the binary toxicity outcome was calculated as (23).


(4) $${\text{LLH}}_{k}=\sum_{\text{k}:y_{k}^{r}=1\;}log\left(NTCP_{LKB}^{k,r}\right)+\sum_{\text{k}:y_{k}^{r}=0}log\left(1-NTCP_{LKB}^{k,r}\right)$$

where *r*=1…R is the patient number, $NTCP_{LKB}^{k,r}$ is a value from the predicted distribution and $y_{k}^{r}$ is a value from the actual distribution; the LLH values arise from a summation over all the patients with different outcomes $y_{k}^{r}=1$ or $y_{k}^{r}=0$ , i.e., with and without the defined GI/GU toxicities respectively. Fitting of the NTCP parameters with clinical outcomes was accomplished by adjusting the values of the model parameters until the best estimates that aim to capture the most frequent patterns in the data could be achieved (16, 24). Fits were made separately considering the DVHs from D_P_ and D_A_. The bootstrapping method was used to calculate the 95% confidence intervals (CIs) of the model parameters (25, 26).

Model performance of the derived LKB-NTCP parameters was measured with respect to its calibration results and discriminative ability. Calibration plots were generated using the Hosmer-Lemeshow p-value (p-HL) goodness of fit test, whereby a p-value of >0.05 indicates that similar observed and predicted probability was achieved (27). For binary dependent variables, the observed outcomes were divided into quartiles to attain the observed probabilities and were plotted against the predicted probabilities. Model discrimination was evaluated using the mean area under the receiver operating characteristic curve (AUC) to determine the overall model fit of the predictors with respect to the defined clinical endpoints. An AUC of ≥0.6 and minimum 95% CI ≥0.5 was considered statistically significant (28). Internal validation using the bootstrap method was performed by resampling the original datasets 1000 times and estimating the biological parameters to establish the 95% CIs for model predictions (29). Statistical analyses were conducted using Matlab (MathWorks, Inc., Natick, MA, USA version 8.0) and IBM SPSS statistics (version 26.0, SPSS, Inc., Chicago, IL).

## Results

Toxicity grading based on the radiation therapy oncology group (RTOG) grading criteria were reviewed and re-graded in accordance with the National Cancer Institute’s Common Toxicity Criteria for Adverse Events (CTCAE) version 4.03 (30) (**Table 1**). This will be aligned with our current toxicity grading system. The investigated clinical endpoints were mild-severe (Grade ≥1) GI and GU, and moderate-severe (Grade ≥2) GI and GU toxicities.

**Table 1:** Late toxicity profiles of 150 patients with HR-PCa.

Gastrointestinal, GI	Grade	N=150, Frequency (%)
Diarrhoea	1	2 (1.3)
	2	2 (1.3)
	3	0 (0)
Proctitis	1	23 (15.3)
	2	9 (6)
	3	0 (0)
Rectal bleeding	1	32 (21.3)
	2	12 (8)
	3	1 (0.7)
Overall maximum	Grade 0	93 (62)
	Grade ≥1	57 (38)
	Grade 0-1	134 (89.3)
	Grade ≥2	16 (10.7)
Genitourinary, GU	Grade	N=150, Frequency (%)
Urinary frequency	1	20 (13.3)
	2	6 (4)
	3	0 (0)
Urinary urgency	1	14 (9.3)
	2	1 (0.7)
	3	0 (0)
Urinary incontinence	1	16 (10.7)
	2	3 (5.3)
	3	0 (0)
Cystitis	1	15 (10)
	2	15 (10)
	3	1 (0.7)
Overall maximum	Grade 0	79 (52.7)
	Grade ≥1	71 (47.3)
	Grade 0-1	125 (83.3)
	Grade ≥2	25 (16.7)

The frequency is shown as the number of patients and the percentage is displayed in parenthesis.

**Figure 1:**
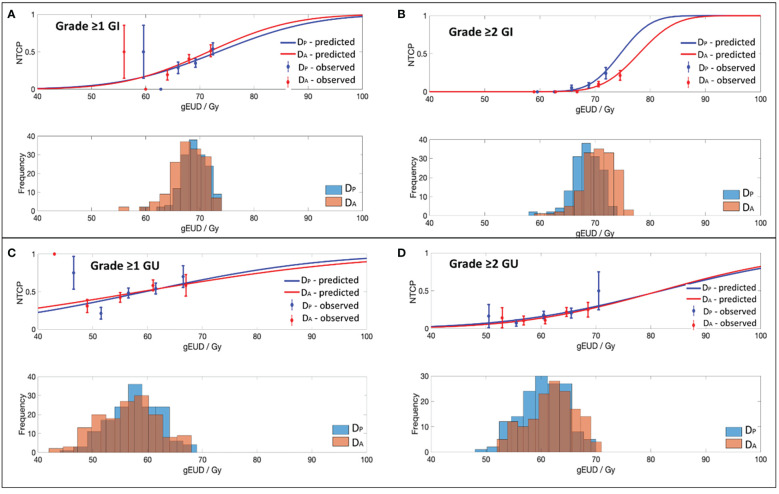
Dose-volume response curves and histograms obtained with the best-fit parameters and 95% CI for the EUD-based NTCP model for **(A)** Grade ≥1 GI, **(B)** Grade ≥2 GI, **(C)** Grade ≥1 GU and **(D)** Grade ≥2 GU toxicity.

**Figure 2:**
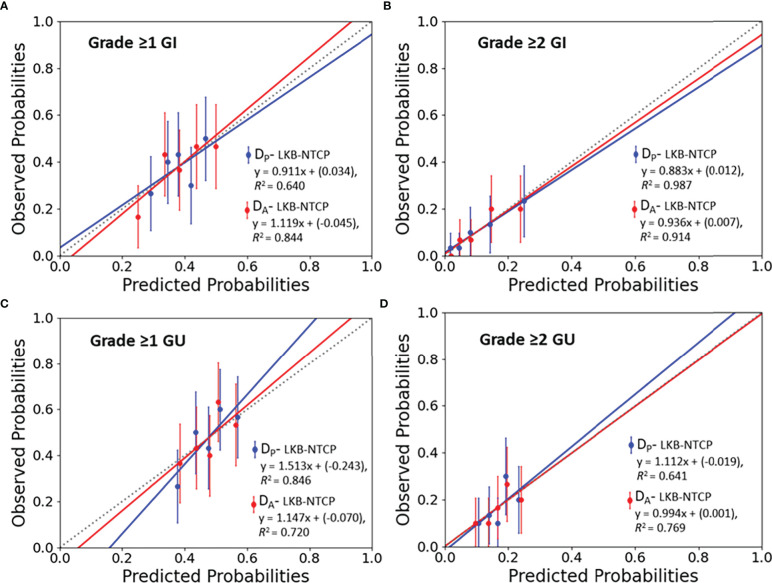
Calibration plots (predicted vs observed probabilities) for LKB-NTCP derived parameters with the associated late toxicity endpoints for **(A)** Grade ≥1 GI, **(B)** Grade ≥2 GI, **(C)** Grade ≥1 GU and **(D)** Grade ≥2 GU toxicity. The 45° dotted line represents the reference line y=x.

Best-fit LKB parameters with the associated 95% CIs and model performance results from using either the D_P_ or D_A_ data were correlated with the defined toxicity endpoints (see **Table 2** and **Figure 1**). Goodness-of fit-test (HL-p value: 0.10 – 0.95) indicated that the model fitted the data adequately while the calibration plots (see **Figure 2**) showed good agreement between the predicted and observed morbidities.

**Table 2:** Biological parameters for LKB-NTCP model fitted with clinical endpoints using D_P_ and D_A_ values and associated model performance results.

LKB-NTCP						Model performance	
Dose	Toxicity	Grade	D_50_, Gy (95% CI)	m (95% CI)	n (95% CI)	HL-p value	AUC (95% CI)
**D_P_**	GI	≥1	73.4 (64.0, 120.1)	0.19 (0.08, 1.6)	0.04 (0.004, 2.0)	0.51	0.58 (0.48, 0.67)
**D_A_**	GI	≥1	71.6 (62.0, 84.1)	0.17 (0.09, 0.75)	0.04 (0.01, 0.20)	0.74	0.62 (0.52, 0.70)
**D_P_**	GI	≥2	74.6 (69.1, 85.4)	0.06 (0.03, 0.14)	0.04 (0.02, 0.09)	0.52	0.75 (0.60, 0.88)
**D_A_**	GI	≥2	77.9 (76.4, 79.6)	0.07 (0.04, 0.15)	0.02 (0.01, 0.07)	0.95	0.73 (0.61, 0.84)
LKB-NTCP						Model performance	
Dose	Toxicity	Grade	D_50_, Gy (95% CI)	m (95% CI)	n (95% CI)	HL-p value	AUC (95% CI)
**D_P_**	GU	≥1	59.5 (44.9, 77.3)	0.43 (0.13, 2.0)	0.19 (0.02, 2.0)	0.10	0.59 (0.50, 0.69)
**D_A_**	GU	≥1	58.8 (44.6, 81.2)	0.56 (0.10, 1.9)	0.21 (0.02, 2.02)	0.64	0.59 (0.50, 0.69)
**D_P_**	GU	≥2	81.9 (68.1, 123.1)	0.27 (0.09, 0.68)	0.14 (0.02, 2.0)	0.63	0.61 (0.48, 0.73)
**D_A_**	GU	≥2	81.7 (69.0, 121.9)	0.24 (0.11, 0.62)	0.12 (0.02, 2.0)	0.87	0.61 (0.48, 0.72)

LKB-NTCP, Lyman-Kutcher-Burman-normal tissue complication probability; D_P_, planned dose; D_A_, accumulated dose; HL-p, Hosmer-Lemeshow p-value; AUC, area under the receiver operating characteristic curve.

### LKB-NTCP parameters for Grade ≥1 GI toxicity

For Grade ≥1 GI toxicity, the D_A_-NTCP model achieved a lower D_50_ value when compared with D_P_-NTCP (D_A_-D_50_: 71.6 Gy vs D_P_-D_50_: 73.4 Gy). Both models obtained similar m values and have the same low n values (n= 0.04) (see **Table 2**). In terms of model performance, although both models obtained a HL-p values of > 0.05, the LKB-NTCP parameters generated using the D_P_ DVH data has a mean AUC of 0.58 (<0.6) and a minimum 95% CI ≤ 0.5. This indicates that the generated LKB-NTCP parameters are less robust in predicting the occurrence of Grade ≥1 GI toxicity events.

### LKB-NTCP parameters for Grade ≥2 GI toxicity

For Grade ≥2 GI toxicity, the obtained D_50_ dose was higher than the reported D_50_ in determining Grade ≥1 GI toxicity in this study. In D_P_-NTCP model, the dose obtained for D_50_ was lower as compared to the D_50_ dose generated from the D_A_-NTCP model (D_P_-D_50_; 74.6 Gy vs D_A_-D_50_; 77.9 Gy). The n value for both models were generally low, but D_A_ generated a slightly lower n value than the D_P_-NTCP model. Both models demonstrated good model performance, whereby a HL-p value of >0.05 and a high AUC of > 0.7 were attained (see **Table 2**). This demonstrated good model fitting results achieved from the predicted and actual clinical toxicity events.

### LKB-NTCP parameters for Grade ≥1 GU toxicity

For Grade ≥1 GU toxicity, D_P_-NTCP has achieved a slightly higher D_50_ (D_P_-D_50_: 59.5 Gy vs D_A_-D_50_: 58.8 Gy), but a slightly lower n value as compared to D_A_-NTCP model (D_P_-n: 0.19 vs D_A_-n: 0.21). A large 95% CIs were observed for the D_50_ parameter. The overall model performance for D_P_-NTCP and D_A_-NTCP generated parameters were slightly inferior as a lower AUCs (<0.6) were observed although the HL-p values for both models were > 0.05 (see **Table 2**).

### LKB-NTCP parameters for Grade ≥2 GU toxicity

The derived D_50_ using both D_P_ and D_A_ DVH data achieved similar values (D_P_-D_50_: 81.9 Gy vs D_A_-D_50_: 81.7 Gy) whilst the achieved n value for D_P_ (D_P_-n:0.14 vs D_A_-n: 0.12) was slightly higher as compared to D_A_. Both D_P_-NTCP and D_A_-NTCP models displayed good model performance by attaining HL-p values of >0.05 and an AUC of >0.6 (see **Table 2**).

## Discussion

The results of this study show the biological parameters obtained based on the clinical observations using modern RT techniques and regimens have a high predictive power in defining the potential occurrence of late GI and GU toxicities. The derived LKB-NTCP parameters were based on D_P_ and D_A_ DVH, thereby taking into consideration the impact of organ variation on the defined clinical toxicity endpoint. Apart from reporting the derived LKB-NTCP parameters based on Grade ≥2 GI and GU toxicities, Grade ≥1 (mild-severe) toxicity was also investigated. This is in parallel with the ongoing efforts in improving patient’s quality of life (QoL) after cancer treatment.

### LKB-parameters for Grade ≥1 GI toxicity

The D_50_ value obtained in this study using either D_P_ or D_A_ DVH data shown that a higher dose range (> 70 Gy) is indicative of patient having Grade ≥1 GI toxicity following RT. A very low n value achieved for both D_P_ and D_A_ (D_P/A_-n: 0.04) implies that the rectum is a serial structure, whereby mild-severe GI toxicity can be caused by high dose being delivered to a very small rectal volume (17). The D_50_ attained using D_A_ DVH has a narrower 95% CI and has achieved a higher mean AUC (> 0.6) as compared to the D_P_ generated NTCP parameters. D_A_ generated NTCP parameters therefore has a higher predictive power in determining the occurrence of Grade ≥1 GI toxicity. Correspondingly, the study conducted by Ospina et al. (31) on 261 prostate cancer patients treated using 3DCRT reported that having a D_50_ of >70 Gy (D_50_: 79.11 Gy; 95% CI: 77.24 - 81.12) and a low n value (n: 0.003; 95% CI: 0.0003-0.0118) were highly predictive for patient having Grade ≥1 rectal bleeding. On the contrary, Gulliford et al. (28) performed GI toxicity fitting using LKB model on 388 prostate cancer patients treated using either a 3-4 fields 3DCRT or 7 fields forward planning technique reported a much lower D_50_ (D_50_: 59.2 Gy; 95% CI: 57.8-61.9 Gy) but with a similar high n value (n:0.14; 95% CI: 0.09-0.16). Differences were observed between the published data and the reported results from this study, as generated NTCP parameters in this study accounted for the variations in organ motion during treatment. Thus, the achieved results will be more applicable in predicting the occurrence of mild-severe GI toxicity in HR-PCa patients using VMAT.

### LKB-NTCP parameters for Grade ≥2 GI toxicity

D_A_-NTCP generated parameters in defining Grade ≥2 GI toxicity obtained a higher D_50_ dose in addition to a lower n value as compared to the D_P_-NTCP obtained parameters in this study. These results demonstrated that having a high D_50_ of 77.9 Gy (50% complication probability) to a very small volume (n:0.02) is highly predictive of patients experiencing Grade ≥2 GI late toxicity. This result reiterates the results obtained from our recent publication using DV metrics in defining the same toxicity endpoint (21). The attained results from the study stated that D0.03 cc of the rectal volume exposed to an accumulated dose of ≥78.2 Gy has a high probability of patients developing Grade ≥2 GI toxicity. Incorporating D_A_-DVH data for model fitting in determining Grade ≥2 GI toxicity also generated a very narrow 95% CI for the obtained D_50_ dose (D_A_-D_50_-95%CI: 76.4-79.6 Gy vs D_A_-D_50_-95%CI: 69.1-85.4 Gy) for the obtained D_50_ dose. Optimal data fitting with good discriminative ability between patients having Grade<2 and Grade ≥2 GI toxicity events was accomplished using the D_A_-DVH data.

D_P_-NTCP parameters (D_50_ and n values) defining Grade ≥2 GI toxicity in this study falls within the 95% CIs as proposed by Michalski et al. (32) (D_50_: 76.9 Gy, 95% CI: 73.7-80.1 Gy & n: 0.09, 95% CI: 0.04-0.14). For the D_A_-DVH generated parameters, whereby patients’ daily variations in organ motion were taken into consideration, the obtained D_50_ was higher with a lower n value. This might signify that the actual dose received by the rectum could be lower as compared to planned, therefore a higher D_50_ will be required to elicit the same toxicity endpoint. Correspondingly, Ospina et al. (31) also reported a much lower n values (n: 0.0060-0.0335) in defining Grade ≥2 rectal bleeding, thereby supporting the serial-like behavior of the rectum. A volume-exponent of a small n value with accompanying high D_50_ dose demonstrated the significant impact of the variations in rectal shape along the prostate gland on the corresponding late GI events (23).

### LKB-NTCP parameters for Grade ≥1 GU toxicity

The bladder being a highly distensible organ has posed great challenges in conducting DV analysis and correlating with GU toxicity (33). For Grade ≥1 late GU toxicity, the D_50_ and n values obtained from the D_P_-NTCP and D_A_-NTCP models suggested that a moderate-high dose range delivered to a larger bladder volume might increase the occurrence of the defined toxicity endpoint (34). The similar NTCP parameters achieved using D_A_ and D_P_ DVH might suggest that despite the extensive volumetric variations of the bladder during RT, the impact on the actual dose received in defining Grade ≥1 GU toxicity is similar. From our knowledge, there are limited published studies reporting on the recommended NTCP parameters for Grade ≥1 late GU toxicity. This result was echoed by the study performed by Thor et al. (35) in comparing the associations between planned and accumulated dose generated using twice a week CT images for 38 prostate cancer patients treated using IMRT techniques. The study concluded that the use of either planned or accumulated dose does not affect the rate of urinary events. With the gradual shift of focus on patient’s QoL, the results presented here will be able to assist clinicians in identifying relevant biological parameters responsible of the occurrence of mild-severe late GU toxicity.

### LKB-NTCP parameters for Grade ≥2 GU toxicity

According to the QUANTEC publication on GU toxicity (36), in view of the extensive volumetric changes of the bladder due to variable bladder filling, it was concluded that both the maximum dose and a relatively large irradiation bladder volume (50%) may be associated with Grade ≥3 late GU toxicity. The obtained D_50_ and n values for Grade ≥2 GI toxicity using D_A_ and D_P_ were higher as compared to the n values achieved for Grade ≥1 GU toxicity. This observation suggested that higher dose to a slightly larger volume of the bladder could result in a higher likelihood of having Grade ≥2 GU toxicity. In parallel to Emami et al. (22) recommended LKB-NTCP values, a similar high D_50_ value (Emami D_50_: 80 Gy) was achieved in this study. However, lower n values were attained for both D_P_ and D_A_ DVH data (D_P/A_-n:0.12-0.14) as compared to the Emami generated value (n: 0.5). The n value for GU toxicity has conflicting results based on published studies (24, 37). This study showed that bladder could be more of a serial-like organ given the lower range of achieved n values in defining moderate-severe late GU toxicity. The achieved D_50_ for both D_P_-NTCP and D_A_-NTCP models has a wide 95% CIs, thus indicating that the resultant models were not very stable in discriminating between patients with and without Grade ≥2 GU toxicity. This could be caused by data imbalance due to fewer toxicity events or LKB- NTCP as the dosimetric variable might not be the only driver of late GU toxicity. Studies have suggested that the inclusion of clinical predictors have improved the overall model performance for the defined GU toxicity (24, 38).

This study does have some limitations. Firstly, the collated toxicity profile used was derived from physician-reported toxicity. Due to the low concordance rate between physician- and patient-reported outcomes measures (PROMs), it has been recommended to incorporate PROMs for best care management (39). As of current, limited PROMs data were available due to various challenges such as the inadequate training and education in healthcare professionals, lack of digital platforms, lack of patient corporation among others (40). Moving forward, ongoing efforts have been made to introduce PROMs in study protocols as well as in routine clinical care to build up the library of reliable toxicity profiles to complement physician-reported toxicity for symptom management and prevention (41). Secondly, external validation was not performed. However, internal validation of the models was conducted by bootstrapping and demonstrated optimal predictive ability of the derived models. Incorporation of prospective data for model validation in the future to further enhance the reliability of the parameters and the subsequent use in plan customization will be required.

All reported series on NTCP derived parameters differs in terms of variations in risk stratifications, prescribed dose, treatment techniques and clinical variables and intervention strategies (32). Additionally, different rectal and bladder complications might be the results of various rectal/bladder pathogenic mechanisms as the existing constructed biological models were based on some assumptions and do not consider all involved biological mechanisms (42). Moreover, risk and severity of GI and GU toxicities have been reported to be associated with other factors such as patient’s characteristics, hormonal therapy and the intake of medications (38, 43, 44). Thus, applications of the recommended LKB-NTCP parameters should include similar scenarios to those which the parameters were derived to have a more reliable toxicity estimation (45). The incorporation of motion-inclusive rectum and bladder dosimetric data in determining biological-based parameters help to overcome the various uncertainties of generating NTCP-based parameters as discussed in several publications (23, 43). Predictive power of the D_A_ derived LKB-NTCP parameters reported in this study could minimise the impact of over-estimation of the risk of complications as the parameters were attained based on inverse planning techniques with daily image-guidance as localization (46).

## Conclusion

The achieved NTCP parameters using modern RT techniques and accounting for organ motion differs from QUANTEC reported parameters. D_A_-D_50_ of 77.9 Gy for GI and D_A_/D_P_-D_50_ of 81.7-81.9 Gy for GU demonstrated good predictability in determining the risk of Grade ≥2 toxicities especially for GI derived parameters. Therefore, these parameters could be incorporated as part of the DV planning constraints to guide dose escalation strategies while minimising the risk of toxicity.

## Data availability statement

The original contributions presented in the study are included in the article/supplementary material. Further inquiries can be directed to the corresponding author.

## Author contributions

AO wrote the initial draft of the manuscript. All authors participated in editing and review of the manuscript. All authors contributed to the article and approved the submitted version.
